# Blood-Meal Sources and *Trypanosoma cruzi* Infection in Coastal and Insular Triatomine Bugs from the Atacama Desert of Chile

**DOI:** 10.3390/microorganisms10040785

**Published:** 2022-04-08

**Authors:** Nicol Quiroga, Juana P. Correa, Ricardo Campos-Soto, Esteban San Juan, Raúl Araya-Donoso, Gabriel Díaz-Campusano, Christian R. González, Carezza Botto-Mahan

**Affiliations:** 1 Departamento de Ciencias Ecológicas, Facultad de Ciencias, Universidad de Chile, Santiago 7800003, Chile; nicol.quiroga@ug.uchile.cl (N.Q.); egsanjuan@gmail.com (E.S.J.); cbotto@uchile.cl (C.B.-M.); 2 Facultad de Medicina Veterinaria, Universidad San Sebastián, Concepción 4080871, Chile; juana.correa@uss.cl; 3 Escuela de Ciencias Agrícolas y Veterinarias, Universidad Viña del Mar, Viña del Mar 2572007, Chile; 4 School of Life Sciences, Arizona State University, Tempe, AZ 85287, USA; raul.araya.d@asu.edu; 5 Instituto de Biología, Facultad de Ciencias, Pontificia Universidad Católica de Valparaíso, Valparaíso 2373223, Chile; gabrieldiazcampusano@gmail.com; 6 Instituto de Entomología, Universidad Metropolitana de Ciencias de la Educación, Santiago 7760197, Chile; christian.gonzalez@umce.cl

**Keywords:** *Mepraia parapatrica*, wild *Trypanosoma cruzi* cycle, wild triatomine vector, *Microlophus*, *Abrothrix*, vector-borne disease

## Abstract

*Mepraia parapatrica* is one of the lesser known and less abundant sylvatic triatomine species naturally infected by the protozoan *Trypanosoma cruzi*, the etiological agent of Chagas disease. *M. parapatrica* lives in sympatry with *T. cruzi*-infected rodents, but only birds, reptiles, and marine mammals have been reported as blood-meal sources of this vector species by serology. The distribution range of this kissing bug overlaps with fishers’ settlements and tourist areas, and therefore the study of the blood-meal sources of this triatomine species is relevant. Here, we determined the blood-meal sources of *M. parapatrica* by NGS or standard sequencing from a coastal mainland area and an island in northern Chile, and *T. cruzi* infection by real-time PCR. The blood-meals of. *M parapatrica* included 61.3% reptiles, 35.5% mammals (including humans) and 3.2% birds. Feeding on reptiles was more frequent on the mainland, while on the island feeding on mammals was more frequent. The presence of *T. cruzi*-infected triatomine bugs and humans as part of the diet of *M. parapatrica* in both areas represents an epidemiological threat and potential risk to the human population visiting or established in these areas. Currently there are no tools to control wild triatomines; these results highlight the potential risk of inhabiting these areas and the necessity of developing information campaigns for the community and surveillance actions.

## 1. Introduction

Blood-sucking insects of the subfamily Triatominae (Hemiptera, Reduviidae) participate as vectors of *Trypanosoma cruzi*, the causative agent of Chagas disease [1]. Triatomine bugs acquire *T. cruzi* mainly when taking blood meals from infected hosts, amplifying the parasites in its gut and transmitting them through faeces [2]. Birds and reptiles are considered refractory to *T. cruzi* infection and only mammals are involved in the transmission cycle [3,4]. The three Chilean species of the endemic genus *Mepraia* have been reported as vectors of *T. cruzi* [5,6,7]. *Mepraia gajardoi* and *M. parapatrica* are species inhabiting coastal and insular areas between 18° and 26° S [8,9,10]. Both species are diurnal; they can be found under stones and associated with marine birds, sea mammals and lizards [11]. A serological study showed that, on Pan de Azúcar Island (26° S), *M. parapatrica* fed on marine birds (78%), sea mammals (15%), and reptiles (7%) [12]. In the same island *T. cruzi*-infected rodents (6.1% infection frequency) and triatomine bugs (20.3% infection frequency) have been reported [13]. Other studies have reported *M. gajardoi* populations associated with fishers’ dwellings and *T. cruzi* infection in dogs living nearby, warning about the epidemiological risk of these triatomine bugs due to the proximity of kissing bugs to human settlements, dogs acting as a reservoir and the potential invasion of triatomine bugs in fishers’ coves [14,15]. Pan de Azúcar National Park (PANP hereafter) is a protected area in northern Chile where fishers’ dwellings and tourist activities occur close to *M. parapatrica* populations. This study assesses the blood meal sources in two populations of *M. parapatrica* on an uninhabited island and a mainland site on the Pacific coast, both within the PANP. *Trypanosoma cruzi*-infection in the mainland kissing bug population was also tested. This knowledge is crucial to establish the epidemiological risk that this triatomine species represents to humans visiting or permanently established in these areas.

## 2. Materials and Methods

### 2.1. Study Areas and Insect Colection

**Figure 1:**
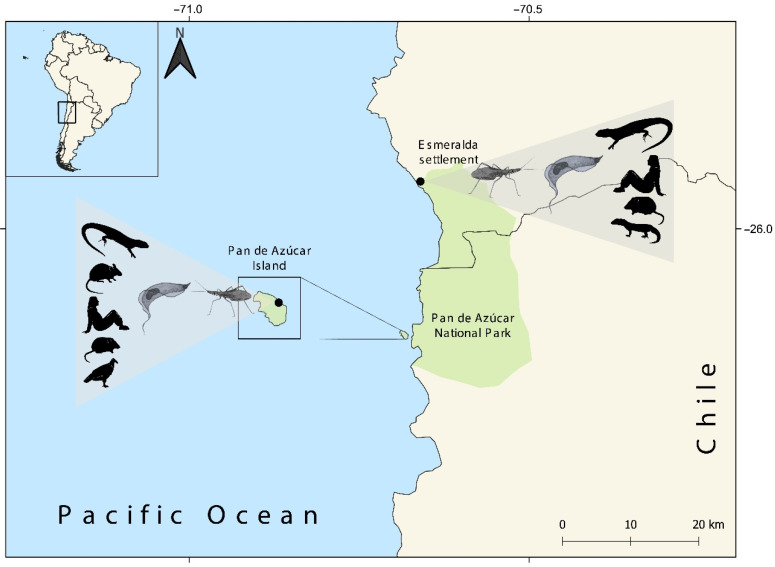
Map of Pan de Azúcar National Park (PANP) off the Pacific coast of southern South America. Black dots represent the study sites in Pan de Azúcar Island and the mainland (Esmeralda settlement). Five vertebrate species were detected in the island blood meal sources (left); from top to bottom: the lizard *Microlophus atacamensis*, the common mouse *Mus musculus*, *Homo sapiens*, the sylvatic mouse *Abrothrix olivaceus* and the vulture *Cathartes aura*. Four species were detected in the mainland blood meal sources (right); from top to bottom: *M. atacamensis*, *H. sapiens*, *A. olivaceus* and the gecko lizard *Garthia gaudichaudii*.

Triatomine bugs were collected in the PANP from Pan de Azúcar Island (island hereafter) and in a site on the mainland coast near the Esmeralda settlement (mainland hereafter) during the austral summer of 2018. Both sites are within the distribution of *M. parapatrica* in arid areas of the Atacama Desert (see map in Figure 1). In each place, two trained people acting as bait manually captured all approaching triatomine bugs for 2.5 h [16]. A total of 87 specimens were collected, 58 in island and 29 on the mainland.

### 2.2. Infection and Cyt b Amplification Analyses

The insects were stored individually and euthanized by chilling. In a cleaned and sterilized working area, bugs were washed with bi-distilled water and then the intestine and its contents were extruded and eluted in 20 μL of ATL buffer (QIAGEN, CA, USA). Whole DNA was extracted using the DNeasy Blood and Tissue Kit (QIAGEN, CA, USA) following manufacturer’s instructions, with a final elution volume of 100 μL. The extracted DNA was stored at −20 °C until molecular analyses. To assess the presence of nuclease inhibitors, an internal amplification control (IAC) of 100 pg of *Arabidopsis thaliana* DNA was added to all the samples during the DNA extraction [17]. For mainland samples, the presence of *T. cruzi* DNA and IAC was detected by real-time PCR using reported protocols [18]. We used a previously reported infection frequency of island kissing bugs detected by real-time PCR [13].

To assess the feasibility of blood meal detection, we performed a real time PCR to detect the presence of vertebrate Cytochrome b (Cyt b) DNA in the triatomine bug samples. For island samples we used those previously collected by [13]. We used the primers Cyt b Fw 5′ CCCCTCAGAATGATATTTGTCCTCA 3′ and Rv 5′CCATCCAACATCTCAGCATGATGAAA 3′ [19] at 0.5 μM and 1× Hot FIREPol ^®^ EvaGreen ^®^ qPCR Mix (ROX) (Solis Biodyne, Estonia) in a final volume of 20 μL. Each sample was tested in duplicate using 2 μL of template vertebrate DNA as positive control and water as non-template control (NTC). The thermal profile was as follows: initial denaturation at 95 °C for 15 min followed by 40 cycles at 60 °C for 20 s and 72 °C for 20 s, finishing with a default melting curve. The samples with cycle threshold below 34 were considered feasible for blood meal identification by standard sequencing or Next Generation Sequencing (NGS hereafter).

### 2.3. Standard and Next Generation Sequencing Analyses

Standard sequencing analysis was performed in Macrogen (Korea) for a sample subset, then sequence quality was evaluated with Proseq v 2.9 [20] and compared to available sequences through BLAST in GenBank. To determine blood meal source we used 97% as the cut-off percentage, but we also included a couple of samples with lower percentage identity (see details in results). We also performed NGS (Figure S1) on three mixtures of 10 or 11 randomly chosen blood-meal samples each, two pools for island and one pool for mainland. We also used NGS on one unidentified mainland sample. We performed a diversity assay using bTEFAP^®^ illumina 20 k on Cyt b of vertebrates, using the same primers described before. The demultiplexed reads were filtered using Sickle 1.33 (https://github.com/ucdavis-bioinformatics/sickle accessed on 28 October 2021), removing reads shorter than 200 bp or with a quality score lower than QV20. We removed adapter and primer sequences with CutAdapt 1.18 [21]. We then used vsearch v2.18.0 [22] to generate operational taxonomic units (OTUs), using 97% sequence identity. OTUs with less than 100 reads of abundance were removed [23]. Then, each OTU was compared to Cyt b sequences from the full NCBI nucleotide database with the Blastn tool, available in https://blast.ncbi.nlm.nih.gov/Blast.cgi (accessed on 29 January 2022). Each OTU was assigned to the species corresponding to the highest Blast score.

### 2.4. Niche Width, Overlap and Statistical Analyses

Cyt b amplification and infection frequency in island and mainland kissing bugs were compared by χ^2^ tests. Niche width, understood as the number or proportion of different species present in the diet of a given species [24,25], was calculated separately for island and mainland kissing bugs as β = 1/(Ʃ*pi*^2^), where *pi* is the relative frequency of prey *i* in the diet [26]. The standard deviation of niche width was estimated by the Jack-knife method [27]; niche width was compared between island and mainland kissing bugs by a *t*-test. Niche overlap was calculated as α = Ʃ*piqi*/√(Ʃ*pi*^2^ Ʃ*qi*^2^), where *pi* and *qi* are the relative frequencies of the prey *i* in the diet of island and mainland kissing bugs, respectively [28]. All statistical analyses were performed with R software (version 3.6.0) with a 95% significance level.

## 3. Results

### 3.1. Infection and Cyt b Amplification 

*Trypanosoma cruzi* infection was detected in 31.0% of triatomine bugs from the mainland. The infection of island triatomine bugs was previously described in [13] and was 20.7%. Comparison between infection frequencies detected in island and mainland kissing bugs did not show a statistically significant difference (χ^2^ = 1.13, *p* = 0.288). IAC were detected in all samples, meaning no inhibitors were present. We detected vertebrate Cyt b amplification in 67.2% and 93.1% of kissing-bugs from the island and mainland, respectively, a statistically significant difference (χ^2^ = 7.06, *p* = 0.008). See complete information in Supplementary Materials Table S1 (database).

### 3.2. Standard Sequencing Analysis

**Figure 2:**
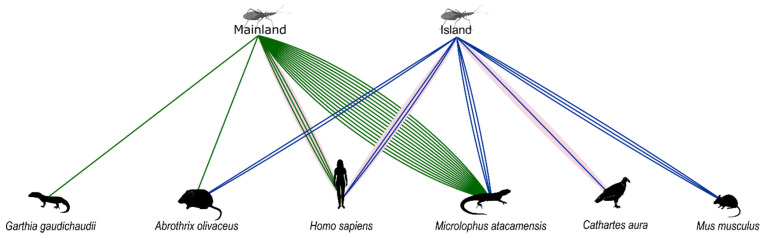
Blood meal sources of *Mepraia parapatrica* in two sites of Pan de Azúcar National Park, mainland (green) and island (blue). Each coloured line corresponds to an individual standard sequencing result. Light pink connections are NGS results. Six vertebrate species were detected; from left to right: the gecko lizard *Garthia gaudichaudii*, the sylvatic mouse *Abrothrix olivaceus*, *Homo sapiens*, the lizard *Microlophus atacamensis*, the vulture *Cathartes aura* and the common mouse *Mus musculus*.

A total of 36 sequences of Cyt b of vertebrates were used for Blast analysis. We detected five vertebrate species by standard sequencing in the island blood-meal sources (the lizard *Microlophus atacamensis*, the common mouse *Mus musculus*, *Homo sapiens*, the sylvatic mouse *Abrothrix olivaceus* and the vulture *Cathartes aura*) and four species in the mainland blood-meal sources (*M. atacamensis*, *H. sapiens*, *A. olivaceus* and the gecko lizard *Garthia gaudichaudii*) (Figure 1). Even though *H. sapiens* (87.73%) and *G. gaudichaudii* (88.94%) exhibited identity percentages lower than the cut-off (97%), we included these species because they were present in the study sites (N. Quiroga, personal observation). Overall blood-meal sources were 61.3% reptiles, 35.5% mammals and 3.2% birds. The most frequent species were *M. atacamensis* and *H. sapiens* (Figure 2). See complete information in Supplementary Materials Table S1 (database) and Table S2 (Blast results).

### 3.3. NGS Analysis 

We obtained 24,369 (pool 1) and 34,686 (pool 2) reads for the analysed samples from island kissing bugs, and 34,959 (pool 3) and 34,929 (one unidentified sample) reads for those from the mainland. Three and two OTUs were identified on the island and the mainland, respectively. We detected *H. sapiens* and *C. aura* on the island, but only *H. sapiens* on the mainland. See complete information in Supplementary Materials Tables S1 and S2 (database).

### 3.4. Niche Width and Overlap Analyses 

Niche widths were 4.481 ± 2.577 and 1.695 ± 3.050 for the island and mainland, respectively. There was a statistically significant difference between the niche widths of the two sites (t = 2.287; *p* = 0.029), indicating that the population of kissing bugs from the island had a larger niche width. The niche overlap between island and mainland feeding profiles was 66.4%.

## 4. Discussion

We detected five vertebrate species in the island blood-meal sources and four species in the mainland blood-meal sources by standard sequencing. Niche widths differed between sites even though niche overlap was 66.4%, probably due to *M. atacamensis* monopolizing most feeding interactions on the mainland. Blood meal sources were 61.3% reptiles, 35.5% mammals and 3.2% birds; the most frequent species were coastal lizards and humans. *Trypanosoma cruzi* infection frequency did not differ between island and mainland kissing bugs, probably because mammals are maintaining the transmission cycle in both areas [13,29]. However, blood-stealing behaviour between kissing bugs could not be discarded as an additional mechanism maintaining *T. cruzi* infection on the island [30]. The most frequent blood meal source in both mainland and island was *M. atacamensis*, 75.0% and 27.3%, respectively, an abundant lizard that shares a similar activity pattern and habitat with *M. parapatrica* [13,31]. This suggests that reptiles could play a role in the *T. cruzi* life-cycle, a hypothesis already suggested for interior valleys [7] and other coastal areas of Chile [13].

*Abrothrix olivaceus*, another detected blood source (5.0% on the mainland and 18.2% on the island), is a native rodent species naturally infected by *T. cruzi* in Pan de Azúcar Island [13]. The presence of *A. olivaceus* in the diet of this vector species highlights its potential role in the transmission cycle of *T. cruzi* in coastal sites, similar to the role described for this rodent species in interior valleys where it coexists with populations of the kissing bug *M. spinolai* [32]. The presence of the invasive rodent *M. musculus* as a blood-meal source (27.3% on the island) was unexpected, since there is no record of its presence on this protected island; therefore, its arrival time and *T. cruzi* infection status remains unknown. Our findings support the presence of a complete *T. cruzi* cycle on Pan de Azúcar Island, where triatomine bugs, rodents and vampire bats are infected by *T. cruzi* [13,29,33].

According to the diet analyses, two other vertebrate species were recorded for the first time. On the island, the scavenger bird species *C. aura*, usually associated with human settlements [34], was recorded in one nymph. On the mainland, the nocturnal lizard *G. gaudichaudii*, which lives under rocks in coastal areas of Chile [35], was also detected in one single nymph.

We detected humans as a blood meal source at both sites (15.0% on the mainland and 18.2% on the island). Although Pan de Azúcar Island is a protected area closed to tourists, it is a nesting site of several marine bird species of interest for researchers and park rangers. The occasional visitors to the island could explain our findings, but also Campos-Soto et al. [8] showed that dispersal of triatomines could occur between insular and mainland sites, and this accidental transportation may explain human blood in the diet of island kissing-bugs. Additionally, NGS detected *H. sapiens* in blood-meal sources, supporting the presence of humans in kissing bug diets from both areas. Humans represent a significant proportion of the diet of other sylvatic triatomine species [36,37,38].

Standard sequencing and NGS studies of blood meal sources of kissing bugs from sylvatic areas might show the complete spectrum of potential hosts maintaining *T. cruzi*, which is useful information for public health and conservation programs [39,40]. In this protected area, mainland populations of *M. parapatrica* are infected with *T. cruzi*, and this area has frequent flows of fishers, alga collectors and tourists, therefore with permanent human presence as with many other coastal areas. We showed that *T. cruzi-*infected *M. parapatrica* are feeding on humans at these sites, which represents a threat to the human population and a potential risk if these populations are vulnerable due to lack of knowledge of the transmission cycle of Chagas disease and its vectors. Our study increases the knowledge of the less-studied and less-abundant *Mepraia* species and supports the concern about Chagas disease risk for human populations in the north-central coast of Chile [14].

## 5. Conclusions

In 1991 the Pan American Health Organization certified Chile as free of *T. cruzi* transmission by the domiciliary vector *Triatoma infestans* and certified this again in 2016. However, the risk of transmission by sylvatic vectors is a reality in the northern coast of Chile, where *Mepraia* species (*M. gajardoi* and *M. parapatrica*) invade the houses of people who occupy these environments or perform recreational activities in the habitats where these wild triatomines occur. In this study we showed that the sylvatic vector *M. parapatrica* feeds on reptiles, humans, rodents and birds. Although *M. parapatrica* is a wild vector, we showed that this triatomine species feeds on humans, which could represent a threat and a potential risk for the human population that lives permanently in or visits these coastal areas. Currently there are no tools to control wild triatomine species adequately, but the threat exists, and government entities should inform communities about the potential risk of inhabiting these areas, developing information campaigns for the community and surveillance actions.

## Data Availability

The data presented in this study are available in the manuscript and in its Supplementary Material (Tables S1 and S2).

## Supplementary Materials

The following supporting information can be downloaded at: https://www.mdpi.com/article/10.3390/microorganisms10040785/s1, Figure S1: Graphical workflow that explain the NGS analysis. Table S1: Database with the information for *Mepraia parapatrica* individuals captured in the Pan de Azúcar National Park, Atacama Region, Chile. Table S2: Detailed sequence assignment of blood meal sources from *Mepraia parapatrica* individuals captured in the Pan de Azúcar National Park, Atacama Region, Chile.
