# Supplementary material for: Blood-Meal Sources and Trypanosoma cruzi Infection in Coastal and Insular Triatomine Bugs from the Atacama Desert of Chile
Source: Microorganisms. 2022 Apr 8;10(4):785. doi: 10.3390/microorganisms10040785 (PMC9028406; doi:10.3390/microorganisms10040785)
Supplement: Supplementary file 1 [file microorganisms-10-00785-s001.zip › microorganisms-1607225-supplementary.pdf]

Figure S1: Graphical workflow that explain the NGS analysis

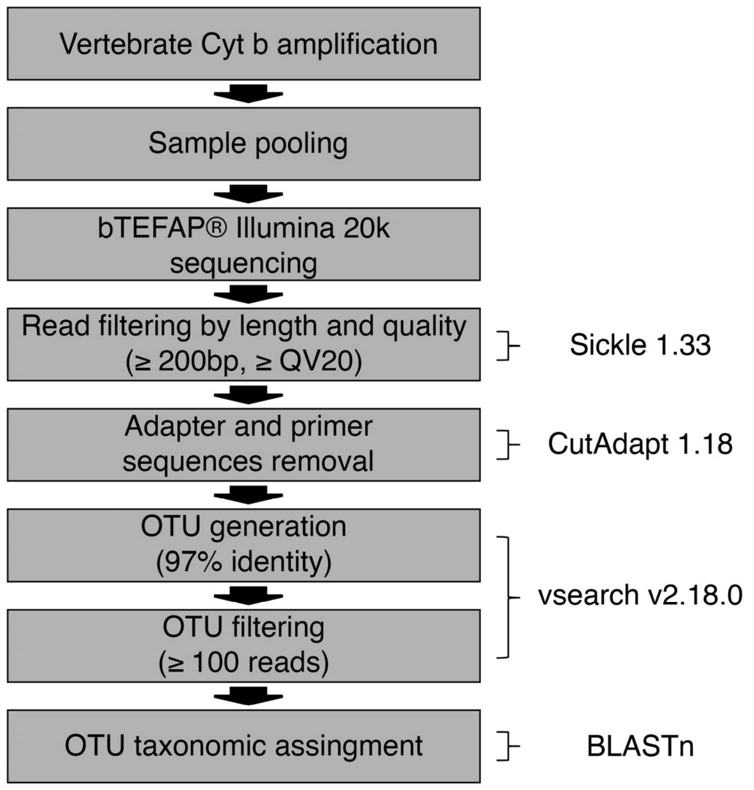

TABLE S1: Database with the information for *Mepraia parapatrica* individuals captured in the Pan de Azúcar National Park, Atacama Region, Chile.

ID: *Mepraia parapatrica* identification number
Sites: Pan de Azúcar Island (Island) or Pan de Azúcar Mainland (Mainland)
Developmental stages: I-V correspond to 1st - 5th stage nymphs, A/F are adult females
Infection Status: Non-infected (0) or infected with *Trypanosoma cruzi* (1)
CytB status: Without vertebrate CytB (0) or with vertebrate CytB in the digestive tract (1)
Sequencing type: Standard sequencing (SS), Next Generation Sequencing (NGS) for samples with Ct < 34
Blood-meal source: Species identified by standard sequencing of individuals or by NGS in three pooled individuals and one single individual (Ma) *Microlophus atacamensis*, (Ca) *Cathartes aurea*, (Hs) *Homo sapiens*, (Mm) *Mus musculus*, (Ao) *Abrothrix olivaceus*, (Gg) *Garthia gaudichaudii,* (NA) non-identified source

ID	SITE	DEVELOPMENTAL STAGE	INFECTION STATUS	CYTB STATUS	SEQUENCING TYPE	BLOOD-MEAL SOURCE
18	Island	I	0	1	-	-
22	Island	I	0	1	SS	Ma
26	Island	I	0	1	-	-
27	Island	I	0	0	-	-
28	Island	I	0	1	-	-
29	Island	I	0	1	-	-
35	Island	I	0	1	SS	Ca
36	Island	I	0	1	-	-
39	Island	I	0	1	-	-
40	Island	I	0	1	-	-
43	Island	I	0	1	SS	NA
44	Island	I	0	1	SS	Ma
45	Island	I	0	0	-	-
46	Island	I	0	1	-	-
47	Island	I	0	0	-	-

49	Island	II	0	1	SS	Hs
50	Island	II	0	1	SS	Ma
51	Island	II	0	1	SS	Mm
52	Island	II	0	1	SS	Mm
53	Island	II	0	1	SS	NA
54	Island	II	0	1	-	-
55	Island	II	0	1	-	-
56	Island	II	0	1	-	-
57	Island	II	0	0	-	-
58	Island	II	0	1	SS	NA
59	Island	II	0	1	SS	Ao
60	Island	II	0	1	SS	Ao
61	Island	III	0	1	SS	Hs
63	Island	III	0	0	-	-
64	Island	III	0	1	-	-
65	Island	III	0	1	SS	NA
66	Island	III	0	0	SS	NA
69	Island	V	0	0	-	-
70	Island	V	0	0	-	-
72	Island	A/F	0	0	-	-
73	Island	A/F	0	0	-	-
19	Island	I	0	1	NGS	
31	Island	I	0	0	NGS	
33	Island	I	0	1	NGS	
37	Island	I	0	1	NGS	
42	Island	I	0	1	NGS	pool-Ca
48	Island	II	0	1	NGS	
62	Island	III	0	0	NGS	
67	Island	III	0	0	NGS	
71	Island	V	0	0	NGS	

74	Island	A/F	0	1	NGS	
17	Island	I	1	1	SS	Mm
16	Island	I	1	1	NGS	
20	Island	I	1	1	NGS	
21	Island	I	1	0	NGS	
23	Island	I	1	0	NGS	
24	Island	I	1	1	NGS	
25	Island	I	1	0	NGS	pool-Hs
30	Island	I	1	0	NGS	
32	Island	I	1	1	NGS	
34	Island	I	1	0	NGS	
38	Island	I	1	1	NGS	
41	Island	I	1	1	NGS	
76	Mainland	I	0	0	-	-
77	Mainland	I	0	1	SS	Ao
80	Mainland	I	0	1	SS	Gg
81	Mainland	I	0	0	-	-
85	Mainland	I	0	1	SS	Ma
87	Mainland	I	0	1	SS	Hs
90	Mainland	II	0	1	SS	Ma
94	Mainland	III	0	1	SS	Ma
95	Mainland	III	0	1	SS	Ma
96	Mainland	IV	0	1	SS	Ma
98	Mainland	IV	0	1	SS	Hs
99	Mainland	IV	0	1	SS	Ma
100	Mainland	V	0	1	SS	Ma
103	Mainland	V	0	1	SS	Ma
75	Mainland	I	0	1	NGS	
78	Mainland	I	0	1	NGS	
79	Mainland	I	0	1	NGS	pool-Hs

84	Mainland	I	0	1	NGS	
86	Mainland	I	0	1	NGS	
88	Mainland	I	0	1	NGS	
82	Mainland	I	1	1	SS	Ma
89	Mainland	II	1	1	SS	Ma
91	Mainland	II	1	1	SS	Ma
92	Mainland	II	1	1	SS	Ma
93	Mainland	II	1	1	SS	Ma
97	Mainland	IV	1	1	SS	Ma
101	Mainland	V	1	1	SS	Hs
102	Mainland	V	1	1	SS	Ma
83	Mainland	I	1	1	NGS	Hs

TABLE S2. Detailed sequence assignment of blood meal sources from *Mepraia parapatrica* individuals captured in the Pan de Azúcar National Park, Atacama Region, Chile.

ISL: Insular site
MNL: Mainland site

SITE	BLOOD MEAL SOURCE	QUERY COVER	E-VALUE	PERCENT IDENTITY	SIZE (BP)	ACCESSION NUMBER
ISL	*Microlophus atacamensis*	100%	9.00E-171	99.70%	334	EF616040.1
ISL	*Microlophus atacamensis*	100%	9.00E-171	99.70%	334	EF616040.1
ISL	*Microlophus atacamensis*	100%	5.00E-168	99.70%	329	EF616040.1
MNL	*Microlophus atacamensis*	100%	2.00E-167	99.70%	328	EF616040.1
MNL	*Microlophus atacamensis*	99%	5.00E-168	99.40%	333	EF616040.1
MNL	*Microlophus atacamensis*	100%	4.00E-164	99.69%	322	EF616040.1
MNL	*Microlophus atacamensis*	100%	3.00E-165	99.69%	324	EF616040.1
MNL	*Microlophus atacamensis*	100%	1.00E-169	99.70%	332	EF616040.1
MNL	*Microlophus atacamensis*	100%	4.00E-169	99.70%	331	EF616040.1
MNL	*Microlophus atacamensis*	100%	3.00E-165	99.69%	324	EF616040.1
MNL	*Microlophus atacamensis*	100%	2.00E-167	99.70%	328	EF616040.1
MNL	*Microlophus atacamensis*	100%	1.00E-168	99.40%	333	EF616040.1
MNL	*Microlophus atacamensis*	100%	2.00E-167	99.70%	328	EF616040.1
MNL	*Microlophus atacamensis*	100%	2.00E-162	99.69%	319	EF616040.1
MNL	*Microlophus atacamensis*	100%	5.00E-168	99.70%	329	EF616040.1
MNL	*Microlophus atacamensis*	100%	2.00E-167	99.70%	328	EF616040.1
MNL	*Microlophus atacamensis*	100%	7.00E-167	99.69%	327	EF616040.1
MNL	*Microlophus atacamensis*	100%	1.00E-164	99.69%	323	EF616040.1
ISL	*Homo sapiens*	100%	1.00E-138	100	274	OL521838.1
ISL	*Homo sapiens*	100%	4.00E-149	97.18%	319	MG660548.1
MNL	*Homo sapiens*	98%	5.00E-83	87.73%	279	KY595576.1
MNL	*Homo sapiens*	100%	4.00E-164	100%	319	MT242596.1
MNL	*Homo sapiens*	100%	4.00E-164	99.09%	328	OL521838.1
ISL	*Abrothrix olivaceus*	100%	2.00E-161	97.91%	335	KF234146.1

ISL	*Abrothrix olivaceus*	98%	9.00E-161	98.48	332	KF234146.1
MNL	*Abrothrix olivaceus*	98%	9.00E-161	98.48%	332	KF234146.1
ISL	*Mus musculus*	100%	2.00E-167	99.70	328	MN627229.1
ISL	*Mus musculus*	98%	3.00E-130	99.62	274	MN964117.1
ISL	*Mus musculus*	100%	6.00E-122	96.67%	270	MN964117.1
ISL	*Cathartes aura*	100%	1.00E-158	97.60%	333	AY509691.1
MNL	*Garthia gaudichaudii*	95%	7.00E-97	88.94%	319	FJ985047.1
